# Resting Cerebrovascular Haemodynamics and Dynamic Assessment of Cerebrovascular Function in Polycystic Ovary Syndrome

**DOI:** 10.1111/cen.70061

**Published:** 2025-11-25

**Authors:** Cory T. Richards, Thomas D. Griffiths, Zoe H. Adams, Melissa E. Wright, Saajan Davies, Jack S. Talbot, Lauren Broad‐Thomas, Diego García Esteban, Jessica J. Steventon, Patrice Brassard, Kevin Murphy, Philip E. James, D. Aled Rees, Rachel N. Lord

**Affiliations:** ^1^ Cardiff School of Sport and Health Sciences Cardiff Metropolitan University Cardiff UK; ^2^ School of Education, Health and Science University of Gloucestershire Gloucester UK; ^3^ Cardiff University Brain Research Imaging Centre (CUBRIC), School of Physics and Astronomy Cardiff University Cardiff UK; ^4^ Cardiometabolic Health and Exercise Physiology Laboratory Baker Heart and Diabetes Institute Melbourne Australia; ^5^ Baker Department of Cardiometabolic Health, Melbourne Medical School University of Melbourne Melbourne Australia; ^6^ Cardiff and Vale University Health Board Cardiff UK; ^7^ Department of Kinesiology, Faculty of Medicine Université Laval Québec Canada; ^8^ Research center of the Institut Universitaire de Cardiologie et de Pneumologie de Québec‐Université Laval Québec Canada; ^9^ Neuroscience and Mental Health Innovation Institute, School of Medicine Cardiff University Cardiff UK

**Keywords:** Cerebral blood flow, Cerebrovascular Circulation, Doppler Ultrasonography, Magnetic Resonance Imaging, Neurovascular Coupling, Polycystic Ovary Syndrome

## Abstract

**Objective:**

Despite an increased cerebrovascular disease risk, the impact of Polycystic Ovary Syndrome (PCOS) on cerebrovascular haemodynamics and function is unknown. This study characterised cerebrovascular haemodynamics and function in women with PCOS versus healthy controls.

**Design:**

Case‐control study.

**Patients:**

Fifteen women with PCOS (age: 31 ± 6 years; body mass index (BMI): 31.8 ± 5.7 kg/m^2^) and 16 controls (age: 30 ± 7 years; BMI: 29.9 ± 5.5 kg/m^2^).

**Measurements:**

Resting global cerebral blood (CBF) was assessed by 3T MRI. Middle‐ and posterior cerebral artery blood velocities (MCAv, PCAv) were measured by Doppler ultrasound and pulsatility index (MCA_PI_, PCA_PI_) calculated. Neurovascular coupling (NVC), internal carotid artery cerebrovascular reactivity (CVR_CO2_) and dynamic cerebral autoregulation (dCA) directional sensitivity were assessed using a visual stimulus, 6% fixed‐inspired CO_2_ and repeated squat‐stand manoeuvres, respectively.

**Results:**

Resting CBF (PCOS: 57.2 ± 7.5 ml/100 g/min; controls: 61.6 ± 11.6 ml/100 g/min, *p* = 0.25) and MCAv, PCAv, MCA_PI_ and PCA_PI_ (all *p* > 0.05) were similar between groups. NVC (14 ± 4.9% vs. 13 ± 3.4%, *p* = 0.45), CVR_CO2_ (5.1 ± 1.9% vs. 6.5 ± 2.9%, *p* = 0.20) and dCA directional sensitivity were similar between groups. However, women with PCOS had elevated relative PCA_PI_ during NVC (PCOS: 12.0 ± 5.6% vs. controls: 7.0 ± 3.8%, *p* = 0.04), and impaired vasodilation of the internal carotid artery during CVR_CO2_ (PCOS: −0.10 ± 0.22 mm vs. controls: 0.18 ± 0.24 mm, *p* < 0.01).

**Conclusions:**

Cerebrovascular function is largely preserved in women with PCOS, although elevated arterial pulsatility and impaired vasodilatory response to carbon dioxide may indicate early endothelial dysfunction in the cerebral vasculature. Larger studies are needed to confirm this in view of our limited study power.

## 1 Introduction

Polycystic Ovary Syndrome (PCOS) is a common endocrine disorder characterised by hyperandrogenism, oligo‐/amenorrhoea, and polycystic ovarian morphology [1]. In addition to its effects on fertility, PCOS is now accepted as a metabolic disorder associated with an increased risk of cardiovascular disease [2], driven by risk factors that include body weight gain [3], insulin resistance [4], arterial hypertension [5], arterial stiffness [6], and endothelial dysfunction [7]. PCOS is also associated with an increased risk of cerebrovascular disease [8], but the cause is unclear. Neuroimaging studies have demonstrated a higher prevalence of markers of cerebral small vessel disease in women with PCOS [9, 10, 11, 12]. Whether these markers reflect clinically relevant reductions in cerebral blood flow (CBF) in PCOS is currently unclear, since previous studies have been limited by measurement of CBF using Doppler ultrasound [13, 14].

CBF and metabolism are tightly coupled in humans to ensure adequate supply to match metabolic demand, and CBF is controlled predominantly via neural, chemical, and autoregulatory factors [15]. Dynamic control of CBF, including neurovascular coupling (NVC) and cerebrovascular reactivity to carbon dioxide (CVR_CO2_), is impaired in related metabolic conditions [16, 17, 18]. These deficits in the dynamic control of CBF may relate to endothelial dysfunction in the cerebral vessels, possibly due to insulin resistance [19]. Hyperandrogenism may also impair cerebrovascular function, since androgens promote vasoconstriction and reduce cerebrovascular responsiveness to elevations in blood pressure in rodent models [20, 21], and increase cerebrovascular pulsatility in postmenopausal women [22]. Women with PCOS may therefore have androgen‐mediated reductions in autoregulatory control of CBF. To date, dynamic regulation of CBF in PCOS has only been assessed in a single study, where the authors inferred that CVR_CO2_ was reduced; however, standard indexes of CVR_CO2_ were not reported, and cohorts were poorly matched [23]. Since impaired CVR_CO2_ and NVC are associated with all‐cause mortality [24] and future cardiovascular disease risk [25], respectively, an understanding of dynamic cerebrovascular responses in women with PCOS has implications for the management of their long‐term health.

We hypothesised that resting CBF and dynamic cerebrovascular control of blood flow, including NVC, CVR_CO2_, and dynamic cerebral autoregulation (dCA), would be reduced in women with PCOS. We investigated this using a comprehensive set of measures to interrogate control of CBF at rest and during dynamic tests of cerebrovascular function.

## 2 Materials and Methods

### 2.1 Participants

Women with PCOS (*n* = 15), diagnosed by the Rotterdam criteria, were recruited via endocrine clinics at the University Hospital of Wales, a PCOS charity (Verity), and social media. Participants with congenital adrenal hyperplasia, Cushing's syndrome, thyroid disease, hyperprolactinaemia, and androgen‐secreting tumours were excluded by biochemical testing. Female controls were matched for age and BMI (*n* = 16) and recruited through social media and word of mouth. PCOS and control participants did not meet recommended physical activity guidelines of 150 min of moderate, or 75 min of vigorous physical activity per week [26], and were excluded if they were pregnant or breastfeeding, had a known history of cardiovascular disease, or had any absolute contraindications to MRI. Participants taking insulin‐sensitisers, oral contraceptives (OC), or anti‐depressants were required to be on a stable dose for at least 3 months. Control participants were excluded if they had clinical or biochemical evidence of hyperandrogenism (*n* = 1). The study was carried out in accordance with the principles of the Declaration of Helsinki, received NHS Research Ethics Committee (REC:19/WA/0093) and institutional ethical approvals (Cardiff Metropolitan University: STA‐9540; Cardiff University: EC.19.12.10.5916 A), and was registered on ClinicalTrials. gov (NCT05394935). All participants gave written, informed consent.

### 2.2 Anthropometric and Biochemical Measurements

Height, body mass, and hip and waist circumference were measured according to our previously published protocol [27]. Blood samples were collected following an overnight fast. Total testosterone and androstenedione were measured by liquid chromatography‐tandem mass spectrometry (Quattro Premier XE triple quadrupole mass spectrometer). Sex hormone binding globulin (SHBG) was measured using an Alinity i immunoassay (Abbott Diagnostics, Illinois, USA). Glucose, total cholesterol, HDL‐cholesterol, LDL‐cholesterol, and triglycerides were measured using an Alinity c immunoassay (Abbott Diagnostics, Illinois, USA). Insulin was measured using an immunometric assay specific for human insulin (Iso‐Insulin ELISA, Mercodia, Sweden). Free androgen index (FAI) was calculated as Testosterone/SHBG*100. The homoeostatic model assessment method (HOMA2‐IR) was used to estimate fasting insulin resistance [28]. The coefficient of variation (CoV%) of all immunoassays was < 3.1%. CoV% of tandem mass spectrometry was 3%–7%–9.5%.

### 2.3 MRI Assessment

Brain MRI data were acquired using a Siemens MAGNETOM Prisma (Siemens Healthcare GmbH, Erlangen; 80 mT/m) 3 T scanner in 15 PCOS and 12 control participants to determine global grey‐matter CBF (gmCBF), in line with our previous work [29]. See Supporting material for MRI acquisition methods, data processing, and analysis.

### 2.4 Cerebrovascular Assessments

#### 2.4.1 Instrumentation

Participants were fitted with a fixed headband, where two 2 MHz transcranial Doppler ultrasound (TCD) transducers (Spencer Technologies, Seattle, WA) measured middle cerebral artery (MCA) and posterior cerebral artery (PCA) blood velocity (MCAv, PCAv) in accordance with guidelines [30]. Sonographer (CTR) CoV% was 3.0% for MCAv and 3.1% for PCAv. Beat‐by‐beat systolic blood pressure (SBP), diastolic blood pressure (DBP), and mean arterial pressure (MAP) were measured via height‐corrected finger arterial clamping (NIBP, AD Instruments, NSW, Australia). Stroke volume (SV) and cardiac output (Q) were estimated using the Windkessel Model. Total arterial compliance was calculated using the SV over pulse pressure method. Pulsatility index (PI) of intracranial arteries (MCA_PI_ and PCA_PI_) was calculated as (peak systolic velocity – minimum diastolic velocity)/mean velocity. Cerebrovascular conductance (CVC) of the intracranial arteries (MCA_CVC_ and PCA_CVC_) was calculated as mean velocity/MAP. All data were collected (Labchart Pro, V8.19, AD Instruments, New Zealand) and saved for later offline analysis. Heart rate (HR) was measured using a Polar monitor (H10, Polar Electro, Finland). Inspired (V'O_2_ and V'CO_2_) and expired respiratory gases (P_ET_CO_2_ and P_ET_O_2_), and minute ventilation (V'E) were measured using an automated breath‐by‐breath offline gas analyser (Vyntus, Vyaire Medical, Illinois, USA).

#### 2.4.2 Dynamic Cerebral Autoregulation Directional Sensitivity

dCA is defined as the ability of the cerebrovasculature to respond to transient increases and decreases in MAP [31]. Accumulating evidence suggests that dCA has directional sensitivity, where changes in cerebral blood velocity are attenuated when MAP increases compared with when MAP decreases [32]. Participants completed 5‐min sets of counterbalanced repeated squat‐stand manoeuvres at 0.10 Hz (5 s) and 0.05 Hz (10 s). This technique provides an insight into the pressure‐flow relationship between the cerebral vasculature and MAP, by forcing cyclic oscillations in MAP. Following a period of standing, participants were instructed to isometrically hold a 90° knee flexion angle during the squat phase, then stand for the same duration (5 s or 10 s for each assessment, respectively). MCAv was collected in participants with PCOS (0.05 Hz, *n* = 14; 0.10 Hz, *n* = 14) and matched controls (0.05 Hz, *n* = 15; 0.10 Hz, *n* = 16). All data were visually inspected for noise before analysis. Data were excluded from one control participant because of insufficient blood pressure recordings. Directional sensitivity methods were used to analyse each repeated squat‐stand protocol [31]. Relative change in MCAv over time, divided by relative change in MAP over time, was calculated for both increases and decreases in MAP (%MCAv_T_/%MAP_T_). For step‐by‐step methodological guidance, see Labrecque, and colleagues [31].

#### 2.4.3 Neurovascular Coupling

NVC is the matching of CBF to metabolic demand in the brain [33]. Using TCD, we measured the PCAv response (a surrogate for CBF response) to an increase in neural demand in the cerebral cortex, elicited by a visual stimulus. Participants completed the NVC assessment following 5 min of seated rest within a darkened room. TCD was used to insonate the PCA. An iPad (iPad Air, Apple, Republic of Ireland, 19 cm x 15 cm display) was placed at eye level 30–35 cm from the participant. This displayed visual stimulation (alternating checkerboard, 2 Hz) to increase neural demand localised to the occipital lobe when participants opened their eyes and fixated on the visual stimulus [33]. Participants completed a baseline 2‐min period of ‘eyes closed’, followed by five cycles of 40 s of ‘eyes open’, focusing on the visual stimulation, preceded by 20 s of ‘eyes closed’. Throughout the protocol, PCAv, PCA_CVC_, PCA_PI_, HR, MAP, P_ET_CO_2_ and P_ET_O_2_ were collected. Changes from baseline to peak response in PCAv, PCA_CVC_ and PCA_PI_ were expressed in relative terms (%). Inadequate PCAv recordings collected from participants with PCOS (*n* = 2) and controls (*n* = 3) were excluded from this analysis (see Supporting methods for data processing and quality control).

#### 2.4.4 Cerebrovascular Reactivity to Carbon Dioxide

Cerebrovascular reactivity to carbon dioxide is a homoeostatic function that helps regulate central pH in the brain. Hypercapnia (induced by CO_2_ inhalation) leads to vasodilation of the cerebral blood vessels and a subsequent increase in CBF [15]. To assess this, longitudinal B‐mode ultrasound images were acquired in the right internal carotid artery (ICA) using a 15 MHz multi‐frequency linear array duplex ultrasound transducer (Terason uSmart 3300, Teratech, MA, USA). Following 5‐min of supine rest, ICA peak envelope velocity and diameter were measured, approximately 1.5 cm distal to the carotid artery bifurcation to minimise the impact of turbulent blood flow. ICA blood flow was calculated as: peak envelope blood velocity/2 × [*π* × (0.5 × diameter)^2^] × 60. Sonographer (CTR) CoV% was 5% and within recommended thresholds for ultrasound acquisition [34].

A three‐way non‐rebreathe valve (Hans Rudolph, Shawnee, KS, USA) attached to a mouthpiece allowed for adjustment from ambient air to a gaseous mixture contained in a Douglas bag. A 2‐min resting recording was measured, followed by inhalation of a fixed concentration of 6% CO_2_, 21% O_2_ and N_2_ balance for 4 min. ICA blood flow and diameter, V'E, P_ET_CO_2_, P_ET_O_2_, HR, SBP, DBP and MAP were measured throughout. CVR_CO2_ was calculated as the maximum change in ICA blood flow per mmHg change in P_ET_CO_2_. The change from baseline to peak response during the final 30 s of the assessment for ICA diameter, MAP, HR, V'E, P_ET_CO_2_ and P_ET_O_2_ are expressed as absolute change. Three participants with PCOS and 1 control participant with inadequate image quality were excluded from this analysis. A further 2 participants with PCOS chose not to undergo this assessment. In 1 control participant, inadequate respiratory gases were collected; hence, CVR_CO2_ data from this participant were not included.

#### 2.4.5 Statistical Analysis

Using the Prism GraphPad software (Version 9.5.1), the normal distribution of all outcome variables was assessed using a Shapiro‐Wilk test. Group differences in normally distributed data were assessed using independent samples t‐tests and reported as mean ± SD. Group differences in non‐normally distributed data were assessed using a Mann‐Whitney U test and reported as median [IQR]. A mixed model analysis of variance (ANOVA) was used to assess the effects of within‐, and between‐groups on dCA (%MCAv_T_/%MAP_T_) in response to increases and decreases in MAP during repeated squat‐stand manoeuvres. Statistical significance was set as *p* < 0.05. Sample size was determined based on an anticipated significant increase in MCAv during exercise, as part of a wider study design, which required 14 participants per group with α = 0.05 and *β* = 0.80. Retrospective power calculations are presented in the Supporting data for outcomes of this study.

## 3 Results

### 3.1 Participant Characteristics

Table 1 summarises the participant characteristics. By design, PCOS and controls were matched for age (PCOS: 31 ± 6 years vs. controls: 30 ± 7 years, *p* = 0.68) and BMI (31.8 ± 5.7 kg/m^2^ vs. 29.9 ± 5.5 kg/m^2^, *p* = 0.36). As expected, participants with PCOS had higher androstenedione (*p* = 0.02) and testosterone (*p* = 0.02) concentrations, and free androgen index (FAI) (*p* = 0.01) compared to controls. Resting SBP, DBP, and MAP, lipids, glucose, insulin, HOMA2‐IR, waist and hip circumference, and their ratio were not different between groups (*p* > 0.05; Table 1). Oral contraceptives were taken by 2 controls (13%). Anti‐depressant/anxiety medication was prescribed in 4 PCOS (27%) and 4 control (25%) participants, respectively. Metformin was prescribed to 1 (7%) individual with PCOS. A retrospective sensitivity analysis was conducted to assess the influence of medication on physiological outcome variables. There were no statistically significant differences between individuals taking medication versus those not taking medication (*p* > 0.05 for all variables).

**Table 1:** Anthropometric and biochemical characteristics of the study population.

	PCOS	Control	*p* value
Age (years)	31 ± 6	30 ± 7	0.68
Body mass index (kg/m^2^)	31.8 ± 5.7	29.9 ± 5.5	0.36
Hip circumference (cm)	115 ± 11	108 ± 14	0.12
Waist circumference (cm)	98 ± 11	96 ± 16	0.74
Waist: Hip ratio	0.85 ± 0.06	0.91 ± 0.18	0.28
Androstenedione (nmol/L)	5.3 ± 1.8	3.9 ± 1.3	**0.02**
Sex hormone binding globulin (nmol/L)	41.5 ± 15.7	56.3 ± 40.5	0.20
Testosterone (nmol/L)	1.4 ± 0.5	1.0 ± 0.3	**0.02**
Free androgen index (%)	3.6 ± 1.4	2.4 ± 1.2	**0.01**
Cholesterol (mmol/L)	5.0 ± 0.9	4.5 ± 0.9	0.17
Triglycerides (mmol/L)	1.0 ± 0.4	0.9 ± 0.3	0.24
HDL‐cholesterol (mmol/L)	1.4 ± 0.2	1.4 ± 0.3	0.52
LDL‐cholesterol (mmol/L)	3.1 ± 0.8	2.7 ± 0.7	0.11
Fasting glucose (mmol/L)	4.7 ± 0.5	4.7 ± 0.4	0.97
Fasting insulin (µU/mL)	10.0 ± 6.1	8.5 ± 3.5	0.39
HOMA2‐IR	1.3 ± 0.8	1.1 ± 0.4	0.36

*Note:* Data are reported as mean ± SD in 15 women with PCOS and 16 matched controls.

Abbreviations: HDL, high‐density lipoprotein; HOMA2‐IR, Homoeostatic Model of Insulin Resistance; LDL, low‐density lipoprotein.

### 3.2 Resting Grey Matter Cerebral Blood Flow

Resting gmCBF was not significantly different between women with PCOS and controls (57.2 ± 7.5 ml/100 g/min vs. 61.6 ± 11.6 ml/100 g/min, *p* = 0.25, respectively).

### 3.3 Resting Cardiovascular and Cerebrovascular Haemodynamics

Resting HR, SBP, DBP, MAP, Q and total arterial compliance were similar between PCOS and control participants (Table 2). MCAv (PCOS: 58.3 ± 6.9 cm/s vs. controls: 66.0 ± 8.2 cm/s, *p* = 0.17), PCAv (PCOS: 46.9 ± 8.4 cm/s vs. controls: 48.7 ± 8.4 cm/s, *p* = 0.82), MCA_CVC_, PCA_CVC_, MCA_PI_ and PCA_PI_ were similar between groups (Table 2).

**Table 2:** Cardiovascular and cerebrovascular characteristics at rest.

	PCOS	Control	*p* value
Heart rate (beats/min)	74 ± 11	76 ± 9	0.40
Systolic blood pressure (mmHg)	119 ± 6	120 ± 10	0.79
Diastolic blood pressure (mmHg)	83 ± 5	83 ± 8	0.73
Mean arterial pressure (mmHg)	95 ± 5	95 ± 8	0.85
Cardiac output (L/min)	2.6 ± 0.6	2.7 ± 0.7	0.58
Total arterial compliance (mL/mmHg)	1.0 ± 0.2	1.0. ± 0.2	0.91
MCAv (cm/s)	58.3 ± 6.9	66.0 ± 8.2	0.17
MCA_PI_ (AU)	0.7 ± 0.1	0.8 ± 0.1	0.29
MCA_CVC_ (cm/s/mmHg)	0.6 ± 0.1	0.7 ± 0.1	0.56
PCAv (cm/s)	46.9 ± 8.4	48.7 ± 8.4	0.82
PCA_PI_ (AU)	0.8 ± 0.1	0.8 ± 0.1	0.97
PCA_CVC_ (cm/s/mmHg)	0.5 ± 0.1	0.5 ± 0.1	0.89

*Note:* Data are reported as mean ± SD in 15 women with PCOS and 16 matched controls.

Abbreviations: MCA, middle cerebral artery; MCA_CVC_, middle cerebral artery cerebrovascular conductance; MCA_PI_, middle cerebral artery pulsatility index; MCAv, middle cerebral artery velocity; PCA, posterior cerebral artery; PCA_PI_, posterior cerebral artery pulsatility index; PCAv, posterior cerebral artery velocity; PCA_CVC_, posterior cerebral artery cerebrovascular conductance.

### 3.4 Dynamic Cerebral Autoregulation Directional Sensitivity

For both 0.05 Hz and 0.10 Hz repeated squat‐stand manoeuvres, there was no hysteresis‐like pattern in the cerebral pressure‐flow relationship and no differences in relative %MCAv_T_/%MAP_T_ for increases or decreases in MAP between groups (Table 3).

**Table 3:** Relative directional sensitivity metrics to repeated squat‐stand manoeuvres.

Direction of pressure change	Frequency of repeated squat‐stand manoeuvres	%MCAv_T_/%MAP_T_ PCOS	%MCAv_T_/%MAP_T_ Control	Group (*p* value)	Condition (*p* value)	Group x Condition (*p* value)
Increase	0.05 Hz	1.66 ± 0.5	1.61 ± 0.4	0.82	0.41	0.55
Decrease	0.05 Hz	1.69 ± 0.5	1.80 ± 0.6			
Increase	0.10 Hz	1.89 ± 0.4	1.83 ± 0.3	0.97	0.83	0.55
Decrease	0.10 Hz	1.80 ± 0.5	1.87 ± 0.5			

*Note:* Data are reported as mean ± SD in 14 individuals with PCOS and 15 matched controls. Group = PCOS vs.versus Control; Condition = Mean arterial pressure increase vs.versus mean arterial pressure decrease.

Abbreviation: %ΔMCAv_T_/%ΔMAP_T_, the time‐adjusted percentage change in MCAv divided by the time‐adjusted percentage change in MAP.

### 3.5 Neurovascular Coupling

**Figure 1:**
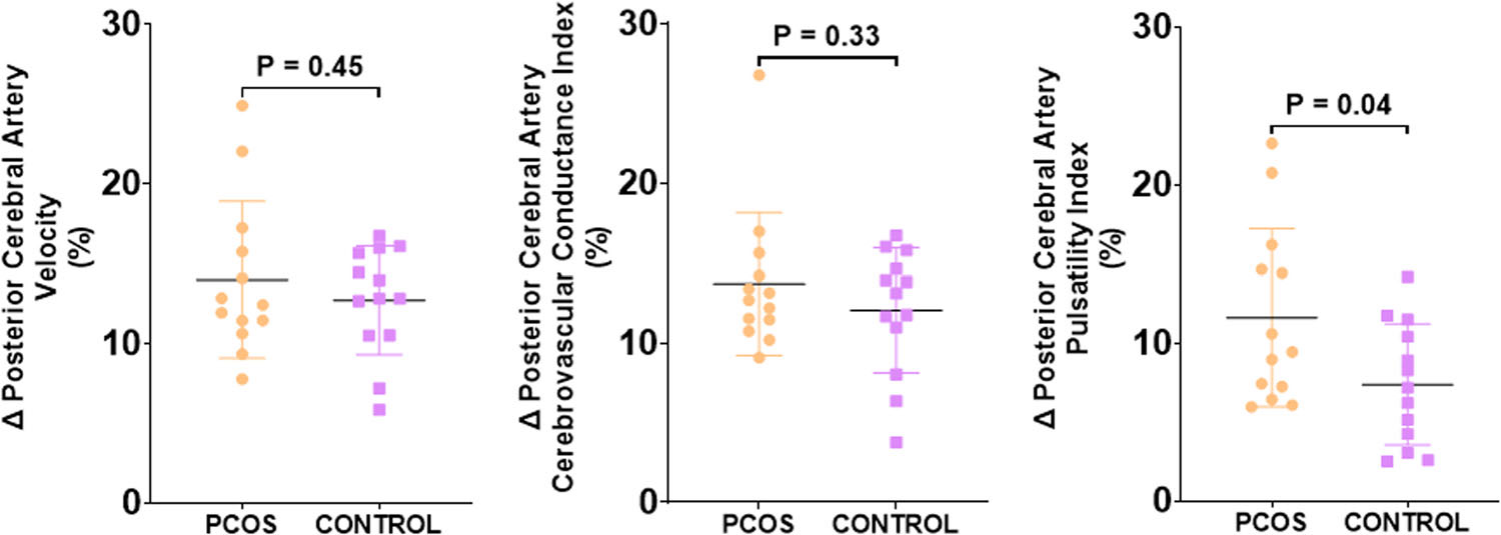
Relative percentage change in posterior cerebral artery velocity (left), cerebrovascular conductance index (middle) and pulsatility index (right) in response to a visual alternating checkerboard stimulus in individuals with PCOS (*n* = 13) and matched controls (*n* = 13).

In response to the visual stimulus, participants with PCOS achieved a similar relative change in PCAv compared with controls (14.0 ± 4.9% vs. 13.0 ± 3.4% respectively, *p* = 0.45). Relative change in PCA_CVC_ was also not different between groups (14.0 ± 4.5% vs. 12.0 ± 3.9%, *p* = 0.33). However, participants with PCOS exhibited a significantly larger relative increase in PCA_PI_ compared with controls (12.0 ± 5.6% vs. 7.0 ± 3.8%, *p* = 0.04) (Figure 1).

### 3.6 Cerebrovascular Reactivity to Carbon Dioxide

**Figure 2:**
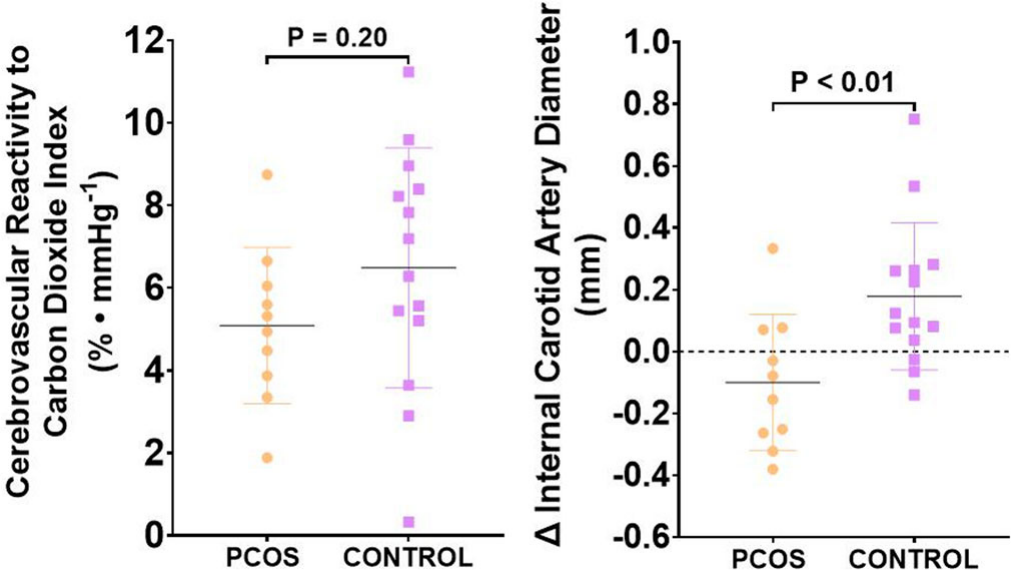
Cerebrovascular reactivity (left) and change in internal carotid artery diameter (right) in response to a 6% fixed inspired concentration of carbon dioxide in individuals with PCOS (*n* = 10) and controls (*n* = 14).

CVR_CO2_ was similar between PCOS and controls (5.1 ± 1.9% vs. 6.5 ± 2.9%, *p* = 0.20) (Figure 2). Absolute changes in MAP, HR, V'E, P_ET_CO_2_ and P_ET_O_2_ were also similar between groups (*p* > 0.05) (Table 4). Absolute change in ICA diameter in response to 6% CO_2_ was lower in PCOS compared with controls (−0.10 ± 0.22 mm vs. 0.18 ± 0.24 mm, *p* < 0.01) (Figure 2).

**Table 4:** Change in haemodynamic and respiratory responses to inhalation of a fixed inspired concentration (6%) of carbon dioxide in women with PCOS and controls.

	PCOS	Control	*p*‐value
Δ Mean Arterial Pressure (mmHg)	3 ± 5	2 ± 4	0.57
Δ Heart Rate (beats/min)	4 ± 5	8 ± 8	0.28
Δ P_ET_CO_2_ (mmHg)	10.6 ± 2.2	10.2 ± 1.8	0.61
Δ P_ET_O_2_ (mmHg)	29.7 ± 7.9	28.2 ± 6.8	0.60
Δ Minute ventilation (L/min)	11.9 ± 4.5	15.3 ± 5.2	0.10

*Note:* Data are reported as mean ± SD in 10 women with PCOS and 15 matched controls.

Abbreviations: P_ET_CO_2_, partial pressure of end‐tidal carbon dioxide; P_ET_O_2_, partial pressure of end‐tidal oxygen.

## 4 Discussion

In this detailed study of cerebrovascular function, we found no evidence of significant alterations in CBF at rest or in response to a series of dynamic challenges in women with PCOS compared to age‐ and BMI‐matched controls. Whilst our findings offer some reassurance that cerebrovascular function is largely preserved in young women with PCOS, our observations of increased PCA pulsatility during NVC and an impaired ICA vasodilatory response to fixed‐inspired carbon dioxide may indicate early endothelial dysfunction in this population.

To our knowledge, this is the first study to provide a comprehensive assessment of cerebrovascular function in women with PCOS, showing that neural, chemical, and autoregulatory cerebrovascular function is preserved. Our findings contrast with the only other study assessing dynamic cerebrovascular responses in PCOS [23], in which an impaired vascular reactivity response to a 5% carbon dioxide stimulus was shown in women with PCOS compared with age‐matched controls. However, Lakhani et al. [23] failed to report the change in mean blood flow (i.e., ‘CVR_CO2_’), and only reported reductions in peak systolic velocity and increased resistance to blood flow. More importantly, participants were not matched for BMI, hence it is conceivable that group differences may have been driven by differences in BMI, rather than PCOS per se, since BMI has in its own right been shown to impair cerebrovascular reactivity to carbon dioxide [35]. Our work extends the findings of Lakhani et al. [23] by assessing the change in ICA blood flow per unit change in P_ET_CO_2_, an index of the reactivity response [30], and aligns with CVR_CO2_ index values in other clinical cohorts with increased risk of cerebrovascular disease, including diabetes and arterial hypertension [36]. However, we found a significantly attenuated ICA diameter response to the CVR_CO2_ assessment in women with PCOS compared with controls, which might indicate a smaller vasodilatory response [19]. In disease states where endothelial dysfunction is apparent, both peripheral and cerebral vascular reactivity to carbon dioxide are impaired [19]. Therefore, while CVR_CO2_ was comparable between groups, the lower ICA diameter response may indicate early endothelial dysfunction in women with PCOS that includes the extra‐cranial arteries. Considering endothelial dysfunction may alter arterial wall structure [37] and can promote atherosclerotic risk [38], our findings may indicate elevated long‐term risk in PCOS. Our study is also the first to examine global gmCBF in women with PCOS using MRI. We observed no differences in gmCBF in PCOS compared with age‐ and BMI‐matched controls, extending previous studies using ultrasound‐derived blood flow measurement of the intra‐ and extra‐cranial arteries [13, 14] by using MRI, widely accepted as the ‘gold standard’ measure. Future studies should aim to integrate assessments of cerebrovascular function, measures of cognitive health, and cerebral microstructure in women with PCOS, given the association between PCOS and altered white‐matter microstructure [10].

Endothelial function plays a pivotal role in the metabolic and chemical control of CBF [19, 33]. Since peripheral endothelial function is impaired in PCOS [7], we anticipated that cerebrovascular function that relies on endothelial‐dependent mechanisms might also be affected. Additionally, in central‐insulin‐resistant rodent models, reduced basal levels of the excitatory neurotransmitter, glutamate, have been observed in the hippocampus compared with controls [39], which may reduce vasodilatory signalling and impair cerebrovascular function. In support of this, NVC [17] and CVR_CO2_ [16, 18] are reduced in diabetes and metabolic syndrome, related disorders in which insulin resistance and endothelial dysfunction are almost universal. In our study, participants with PCOS had similar HOMA2‐IR values as controls, and markedly lower values than typically reported in metabolic syndrome [40] and diabetes [41]. Therefore, it is possible that the degree of insulin resistance in our cohort might have been insufficient to significantly affect cerebrovascular endothelial function. In contrast, as anticipated, we were able to confirm hyperandrogenism in our PCOS group. Testosterone levels have been correlated with impaired endothelial function in women with PCOS, albeit only in lean individuals [42]. Since our study population was overweight (mean BMI 31.8 kg/m^2^), our findings may reflect those of Berbrier and colleagues [42], where the impact of androgens on endothelial function was diminished at a greater BMI. Furthermore, when non‐endothelium‐dependent mechanisms, such as sympathetic activity, are controlled for, endothelial function in the peripheral and cerebral circulation does not appear to be correlated [43]. This may further account for our findings of comparable NVC and CVR_CO2_ responses between groups. Nevertheless, despite a preserved NVC response, and similar PCAv, and PCA_CVC_, we observed a significantly elevated PCA_PI_ in women with PCOS compared with controls in response to the NVC stimulus. Pulsatility index is a measure of arterial stiffening, which has been demonstrated in the peripheral [6] and extra‐cranial vasculature [44] in women with PCOS, and is correlated with hyperandrogenism. Our findings may thus suggest that arterial stiffness in PCOS may extend to the cerebral small vessels but is unmasked only during dynamic stress. As the brain is a high‐flow, low‐impedance end‐organ [45], excessive pulsatile energy transmitted into the cerebral small vessels can cause microvascular damage [45]. Given that cerebrovascular pulsatility index is prognostic of recurrent vascular events [46], cognitive impairment [45, 47], lower grey‐ and white‐matter volumes [45] and cerebral small vessel disease [48], our findings may provide an early indication that the long‐term risk of cerebrovascular disease could be increased in PCOS. However, in our cohort of women with PCOS, cerebrovascular function remains largely intact and clinical implications are likely limited at this stage. In addition, the average age of participants in previous studies on other metabolic disorders differs from our study. Typically, in metabolic conditions, the age of participants spans middle‐aged onwards, reflecting decades of exposure to metabolic dysfunction and its sequelae. In diabetes, CVR_CO2_ in patients within 10 years of diagnosis was significantly greater than those with > 10 years of exposure [49]. Therefore, our population of young women with PCOS may not have been exposed to the metabolic sequelae associated with the condition for long enough to have a detrimental impact on cerebrovascular function.

We hypothesised that dCA directional sensitivity might be impaired in PCOS compared to controls, since androgens have been shown to cause cerebral vasoconstriction and reduced vascular responsiveness to fluctuations in blood pressure in rodent models [20, 21], and increase cerebral vessel pulsatility in postmenopausal women [22]. However, directional sensitivity in the cerebral pressure‐flow relationship was not present in these participants, and %MCAv_T_/%MAP_T_ were similar between groups, reflecting a similar capacity of the MCA to accommodate large fluctuations in MAP. The absence of a hysteresis‐like pattern at 0.10 Hz repeated squat‐stands is surprising, considering that most of the work using this analytical method reported dCA directional sensitivity at this frequency in healthy participants. To our knowledge, this is the first study to assess this method in a clinical population, including overweight subjects. Previous work has highlighted that findings from existing studies may not necessarily translate to clinical populations [50], and it is conceivable that differences in study populations may explain our contrasting findings. Therefore, further research is needed to determine whether a more sensitive response when MAP acutely increases or a less sensitive response when MAP acutely decreases is responsible for the absence of dCA directional sensitivity in these participants.

Our study has several strengths, including a well‐matched control group and the comprehensive assessment of cerebrovascular function in both the resting state and following a series of acute physiological challenges. Nevertheless, our study also had some limitations. First, retrospective power calculations indicated that our study had low power to detect differences between women with PCOS and controls (Supplementary Table 1). Therefore, larger population‐scale studies are necessary to understand whether our findings are generalisable to the wider PCOS population. A further limitation is that we did not adjust for multiple comparisons, potentially increasing the risk of type 1 error; hence, significant results should be interpreted with caution and considered exploratory at this stage. Second, whilst TCD is an inexpensive and noninvasive method of assessing cerebral blood velocity, it is a surrogate marker of CBF and assumes that vessel diameter remains constant. Despite this, TCD provides high‐temporal resolution, enabling the measurement of beat‐by‐beat changes in cerebrovascular function. Third, we included individuals who were currently taking medication (including oral contraceptives, insulin‐sensitizers and anti‐depressants). Whilst such a sample is more representative of the clinical PCOS population at large, in whom contraceptive, insulin‐sensitising and anti‐depressant medication use is common, we acknowledge that such medication might have had an impact on our physiological measures despite mandating a stable dose for at least 3 months before recruitment. Therefore, we cannot clearly conclude that PCOS status alone does not impact cerebral haemodynamics and cerebrovascular function. Future studies should aim to understand these outcomes in medication‐naïve cohorts. Fourth, we recruited individuals with PCOS using the Rotterdam criteria, in keeping with international recommendations [1]. Therefore, our cohort of women with PCOS may represent a milder phenotype of the condition. Nevertheless, it is possible that differences between groups may have been demonstrated had we selected patients with a more severe phenotype, such as that based on NIH criteria, in whom cardiometabolic risk has been shown to be more apparent [51]. Finally, we assessed dCA using the directional sensitivity method [31] and not the traditional method of transfer function analysis (TFA). Therefore, our findings may not be directly comparable to other studies. We believe this technique provides a more valid method of assessing the cerebral pressure‐flow relationship than TFA, given the hysteresis‐like pattern in the cerebral pressure‐flow relationship believed to occur in humans, and that TFA assumes a linear, symmetrical cerebrovascular response to increases and decreases in MAP [52]. While these assessments were conducted in a resting state or following an acute stressor, future work adopting a whole‐body integrated stress challenge, such as exercise, might be anticipated to unmask cerebrovascular dysfunction in this population.

In conclusion, our study demonstrates that individuals with PCOS have largely comparable gmCBF, and cerebrovascular function to age‐ and BMI‐matched controls. Our observations offer some reassurance, with no major differences detected in cerebrovascular function in young women with PCOS. However, longer‐term studies are needed to determine whether the increased arterial pulsatility and impaired carotid vasodilatory responses to carbon dioxide challenge evident in our cohort lead to an increased risk of cerebrovascular events in the future or whether cerebrovascular function becomes impaired in women with PCOS in later life.

## Conflicts of Interest

The authors declare no conflicts of interest.

## Supporting information

Supporting Materials FINAL.

## Data Availability

The data that support the findings of this study are available from the corresponding author upon reasonable request.

## References

[1] H. J. Teede , C. T. Tay , J. J. E. Laven , et al., International PCOS Network ., “Recommendations From the 2023 International Evidence‐Based Guideline for the Assessment and Management of Polycystic Ovary Syndrome,” European Journal of Endocrinology 189, no. 2 (2023): G43–G64, 10.1093/ejendo/lvad096. 37580861

[2] T. R. Berni , C. L. Morgan , and D. A. Rees , “Women With Polycystic Ovary Syndrome Have an Increased Risk of Major Cardiovascular Events: A Population Study,” Journal of Clinical Endocrinology and Metabolism 106, no. 9 (2021): 3369, 10.1210/clinem/dgab392. PMC8372630 34061968

[3] E. Diamanti‐Kandarakis and A. Dunaif , “Insulin Resistance and the Polycystic Ovary Syndrome Revisited: An Update on Mechanisms and Implications,” Endocrine Reviews 33, no. 6 (2012): 981–1030, 10.1210/er.2011-1034. 23065822 PMC5393155

[4] N. K. Stepto , S. Cassar , A. E. Joham , et al., “Women With Polycystic Ovary Syndrome Have Intrinsic Insulin Resistance on Euglycaemic‐Hyperinsulaemic Clamp,” Human Reproduction 28, no. 3 (2013): 777–784, 10.1093/humrep/des463. 23315061

[5] J. R. Mellembakken , A. Mahmoudan , L. Mørkrid , et al., “Higher Blood Pressure in Normal Weight Women With PCOS Compared to Controls,” Endocrine Connections 10, no. 2 (2021): 154–163, 10.1530/EC-20-0527. 33416512 PMC7983477

[6] D. Sun , Y. Wu , M. Ding , and F. Zhu , “Comprehensive Meta‐Analysis of Functional and Structural Markers of Subclinical Atherosclerosis in Women With Polycystic Ovary Syndrome,” Angiology 73, no. 7 (2022): 622–634, 10.1177/00033197211072598. 35258380

[7] V. S. Sprung , G. Atkinson , D. J. Cuthbertson , et al., “Endothelial Function Measured Using Flow‐Mediated Dilation in Polycystic Ovary Syndrome: A Meta‐Analysis of the Observational Studies,” Clinical Endocrinology 78, no. 3 (2013): 438–446, 10.1111/j.1365-2265.2012.04490.x. 22775449

[8] V. Wekker , L. van Dammen , A. Koning , et al., “Long‐Term Cardiometabolic Disease Risk in Women With PCOS: A Systematic Review and Meta‐Analysis,” Human Reproduction Update 26, no. 6 (2020): 942–960, 10.1093/humupd/dmaa029. 32995872 PMC7600286

[9] C.‐A. Castellano , J.‐P. Baillargeon , S. Nugent , et al., “Regional Brain Glucose Hypometabolism in Young Women With Polycystic Ovary Syndrome: Possible Link to Mild Insulin Resistance,” PLoS One 10, no. 12 (2015): e0144116, 10.1371/journal.pone.0144116. 26650926 PMC4674147

[10] D. A. Rees , M. Udiawar , R. Berlot , D. K. Jones , and M. J. O'sullivan , “White Matter Microstructure and Cognitive Function in Young Women With Polycystic Ovary Syndrome,” Journal of Clinical Endocrinology and Metabolism 101, no. 1 (2016): 314–323, 10.1210/jc.2015-2318. 26574952 PMC4701841

[11] Z. Guoqing , S. Fang , D. Lihui , et al., “Cerebral White Matter Lesions and Silent Cerebral Infarcts in Postmenopausal Women With Polycystic Ovary Syndrome,” Gynecological Endocrinology 32, no. 8 (2016): 655–658, 10.3109/09513590.2016.1149812. 26941198

[12] B. Ozgen Saydam , A. C. Has , G. Bozdag , K. K. Oguz , and B. O. Yildiz , “Structural Imaging of the Brain Reveals Decreased Total Brain and Total Gray Matter Volumes in Obese but Not in Lean Women With Polycystic Ovary Syndrome Compared to Body Mass Index‐Matched Counterparts,” Gynecological Endocrinology 33, no. 7 (2017): 519–523, 10.1080/09513590.2017.1295440. 28277117

[13] M. Acar , S. Cevrioğlu , A. Yücel , B. Değirmenci , R. Albayrak , and A. Haktanır , “Evaluation OF Cerebral Blood Flow Volume Using Color Duplex Sonography in Patients With Polycystic Ovary Syndrome,” Electronic Journal of General Medicine 2, no. 3 (2005): 91–95, 10.29333/ejgm/82278.

[14] S. Kizkin , Y. Engin‐Ustun , Y. Ustun , C. Ozcan , S. Serbest , and H. I. Ozisik , “Cerebral Artery Hemodynamics in Polycystic Ovary Syndrome,” Gynecological Endocrinology 21, no. 5 (2005): 287–291, 10.1080/09513590500402848. 16373248

[15] P. N. Ainslie and J. Duffin , “Integration of Cerebrovascular CO_2_ Reactivity and Chemoreflex Control of Breathing: Mechanisms of Regulation, Measurement, and Interpretation,” American Journal of Physiology‐Regulatory, Integrative and Comparative Physiology 296, no. 5 (2009): R1473–R1495, 10.1152/ajpregu.91008.2008. 19211719

[16] S. Giannopoulos , B. Boden‐Albala , J. H. Choi , et al., “Metabolic Syndrome and Cerebral Vasomotor Reactivity,” European Journal of Neurology 17, no. 12 (2010): 1457–1462, 10.1111/j.1468-1331.2010.03087.x. 20500212

[17] A. Canna , F. Esposito , G. Tedeschi , et al., “Neurovascular Coupling in Patients With Type 2 Diabetes Mellitus,” Frontiers in Aging Neuroscience 14 (2022): 976340, 10.3389/fnagi.2022.976340. 36118711 PMC9476313

[18] M. Ivankovic , M. Radman , A. Gverovic‐Antunica , S. Tesanovic , G. Trgo , and V. Demarin , “Influence of Hypertension and Type 2 Diabetes Mellitus on Cerebrovascular Reactivity in Diabetics With Retinopathy,” Annals of Saudi Medicine 33, no. 2 (2013): 130–133, 10.5144/0256-4947.2013.130. 23562999 PMC6078608

[19] S. Lavi , D. Gaitini , V. Milloul , and G. Jacob , “Impaired Cerebral CO_2_ Vasoreactivity: Association With Endothelial Dysfunction,” American Journal of Physiology‐Heart and Circulatory Physiology 291, no. 4 (2006): H1856–H1861, 10.1152/ajpheart.00014.2006. 16766649

[20] G. G. Geary , D. N. Krause , and S. P. Duckles , “Gonadal Hormones Affect Diameter of Male Rat Cerebral Arteries Through Endothelium‐Dependent Mechanisms,” American Journal of Physiology‐Heart and Circulatory Physiology 279, no. 2 (2000): H610–H618, 10.1152/ajpheart.2000.279.2.H610. 10924060

[21] R. J. Gonzales , D. N. Krause , and S. P. Duckles , “Testosterone Suppresses Endothelium‐Dependent Dilation of Rat Middle Cerebral Arteries,” American Journal of Physiology‐Heart and Circulatory Physiology 286, no. 2 (2004): H552–H560, 10.1152/ajpheart.00663.2003. 14551047

[22] M. Penotti , L. Sironi , L. Cannata , et al., “Effects of Androgen Supplementation of Hormone Replacement Therapy on the Vascular Reactivity of Cerebral Arteries,” Fertility and Sterility 76, no. 2 (2001): 235–240, 10.1016/S0015-0282(01)01923-9. 11476766

[23] K. Lakhani , N. Constantinovici , W. Purcell , R. Fernando , and P. Hardiman , “Internal Carotid‐Artery Response to 5% Carbon Dioxide in Women With Polycystic Ovaries,” Lancet 356, no. 9236 (2000): 1166–1167, 10.1016/S0140-6736(00)02764-1. 11030301

[24] M. L. P. Portegies , R. F. A. G. de Bruijn , A. Hofman , P. J. Koudstaal , and M. A. Ikram , “Cerebral Vasomotor Reactivity and Risk of Mortality,” Stroke 45, no. 1 (2014): 42–47, 10.1161/STROKEAHA.113.002348. 24203842

[25] S. Yang and A. J. S. Webb , “Reduced Neurovascular Coupling Is Associated With Increased Cardiovascular Risk Without Established Cerebrovascular Disease: A Cross‐Sectional Analysis in UK Biobank,” Journal of Cerebral Blood Flow & Metabolism 45, no. 5 (2024): 0271678X241302172, 10.1177/0271678X241302172. PMC11585009 39576882

[26] F. C. Bull , S. S. Al‐Ansari , S. Biddle , et al., “World Health Organization 2020 Guidelines on Physical Activity and Sedentary Behaviour,” British Journal of Sports Medicine 54, no. 24 (2020): 1451–1462, 10.1136/bjsports-2020-102955. 33239350 PMC7719906

[27] S. Watson , H. L. Blundell , W. D. Evans , H. Griffiths , R. G. Newcombe , and D. A. Rees , “Can Abdominal Bioelectrical Impedance Refine the Determination of Visceral Fat From Waist Circumference?,” Physiological Measurement 30, no. 7 (2009): N53–N58, 10.1088/0967-3334/30/7/N01. 19436083

[28] J. C. Levy , D. R. Matthews , and M. P. Hermans , “Correct Homeostasis Model Assessment (HOMA) Evaluation Uses the Computer Program,” Diabetes Care 21, no. 12 (1998): 2191–2192, 10.2337/diacare.21.12.2191. 9839117

[29] J. J. Steventon , H. L. Chandler , C. Foster , et al., “Changes in White Matter Microstructure and MRI‐Derived Cerebral Blood Flow After 1‐week of Exercise Training,” Scientific Reports 11, no. 1 (2021): 22061, 10.1038/s41598-021-01630-7. 34764358 PMC8586229

[30] C. K. Willie , F. L. Colino , D. M. Bailey , et al., “Utility of Transcranial Doppler Ultrasound for the Integrative Assessment of Cerebrovascular Function,” Journal of Neuroscience Methods 196, no. 2 (2011): 221–237, 10.1016/j.jneumeth.2011.01.011. 21276818

[31] L. Labrecque , J. S. Burma , M.‐A. Roy , J. D. Smirl , and P. Brassard , “Reproducibility and Diurnal Variation of the Directional Sensitivity of the Cerebral Pressure‐Flow Relationship in Men and Women,” Journal of Applied Physiology 132, no. 1 (2022): 154–166, 10.1152/japplphysiol.00653.2021. 34855525

[32] P. Brassard , L. Labrecque , J. D. Smirl , et al., “Losing the Dogmatic View of Cerebral Autoregulation,” Physiological Reports 9, no. 15 (2021): e14982, 10.14814/phy2.14982. 34323023 PMC8319534

[33] R. L. Hoiland , H. G. Caldwell , C. A. Howe , et al., “Nitric Oxide Is Fundamental to Neurovascular Coupling in Humans,” Journal of Physiology 598, no. 21 (2020): 4927–4939, 10.1113/JP280162. 32785972

[34] K. N. Thomas , N. C. S. Lewis , B. G. Hill , and P. N. Ainslie , “Technical Recommendations for the Use of Carotid Duplex Ultrasound for the Assessment of Extracranial Blood Flow,” American Journal of Physiology‐Regulatory, Integrative and Comparative Physiology 309, no. 7 (2015): R707–R720, 10.1152/ajpregu.00211.2015. 26157060

[35] L. Glodzik , H. Rusinek , T. Butler , et al., “Higher Body Mass Index Is Associated With Worse Hippocampal Vasoreactivity to Carbon Dioxide,” Frontiers in Aging Neuroscience 14 (2022): 948470, 10.3389/fnagi.2022.948470. 36158536 PMC9491849

[36] R. L. Hoiland , J. A. Fisher , and P. N. Ainslie , “Regulation of the Cerebral Circulation by Arterial Carbon Dioxide,” Comprehensive Physiology 9, no. 3 (2019): 1101–1154, 10.1002/cphy.c180021. 31187899

[37] L. Ghiadoni , S. Taddei , A. Virdis , et al., “Endothelial Function and Common Carotid Artery Wall Thickening in Patients With Essential Hypertension,” Hypertension 32, no. 1 (1998): 25–32, 10.1161/01.hyp.32.1.25. 9674633

[38] M. A. Gimbrone and G. García‐Cardeña , “Endothelial Cell Dysfunction and the Pathobiology of Atherosclerosis,” Circulation Research 118, no. 4 (2016): 620–636, 10.1161/CIRCRESAHA.115.306301. 26892962 PMC4762052

[39] J. M. Erichsen , J. L. Woodruff , C. A. Grillo , G. G. Piroli , J. R. Fadel , and L. P. Reagan , “Hippocampal‐Specific Insulin Resistance Elicits Synaptic Effects on Glutamate Neurotransmission,” Journal of Neurochemistry 169, no. 6 (2025): e70083, 10.1111/jnc.70083. 40452372 PMC12127840

[40] K. J. Carter , A. T. Ward , J. M. Kellawan , et al., “Reduced Basal Macrovascular and Microvascular Cerebral Blood Flow in Young Adults With Metabolic Syndrome: Potential Mechanisms,” Journal of Applied Physiology 135, no. 1 (2023): 94–108, 10.1152/japplphysiol.00688.2022. 37199780 PMC10292973

[41] Y. Cui , X. Liang , H. Gu , et al., “Cerebral Perfusion Alterations in Type 2 Diabetes and Its Relation to Insulin Resistance and Cognitive Dysfunction,” Brain imaging and behavior 11, no. 5 (2017): 1248–1257, 10.1007/s11682-016-9583-9. 27714551 PMC5653700

[42] D. E. Berbrier , C. A. Leone , T. E. Adler , et al., “Effects of Androgen Excess and Body Mass Index on Endothelial Function in Women With Polycystic Ovary Syndrome,” Journal of Applied Physiology 134, no. 4 (2023): 868–878, 10.1152/japplphysiol.00583.2022. 36861670

[43] K. Prakash , D. S. Chandran , R. Khadgawat , A. K. Jaryal , and K. K. Deepak , “Correlations Between Endothelial Function in the Systemic and Cerebral Circulation and Insulin Resistance in Type 2 Diabetes Mellitus,” Diabetes & Vascular Disease Research 13, no. 1 (2016): 49–55, 10.1177/1479164115604120. 26408643

[44] K. Lakhani , A. M. Seifalian , and P. Hardiman , “Impaired Carotid Viscoelastic Properties in Women With Polycystic Ovaries,” Circulation 106, no. 1 (2002): 81–85, 10.1161/01.CIR.0000020681.19400.8A. 12093774

[45] G. F. Mitchell , M. A. van Buchem , S. Sigurdsson , et al., “Arterial Stiffness, Pressure and Flow Pulsatility and Brain Structure and Function: The Age, Gene/Environment Susceptibility – Reykjavik Study,” Brain 134, no. 11 (2011): 3398–3407, 10.1093/brain/awr253. 22075523 PMC3212721

[46] A. D. Wijnhoud , P. J. Koudstaal , and D. W. J. Dippel , “The Prognostic Value of Pulsatility Index, Flow Velocity, and Their Ratio, Measured With Tcd Ultrasound, in Patients With a Recent TIA or Ischemic Stroke,” Acta Neurologica Scandinavica 124, no. 4 (2011): 238–244, 10.1111/j.1600-0404.2010.01462.x. 21198447

[47] T. G. Bailey , T. Klein , A. L. Meneses , et al., “Cerebrovascular Function and Its Association With Systemic Artery Function and Stiffness in Older Adults With and Without Mild Cognitive Impairment,” European Journal of Applied Physiology 122, no. 8 (2022): 1843–1856, 10.1007/s00421-022-04956-w. 35522276 PMC9287231

[48] Y. Shi , M. J. Thrippleton , G. W. Blair , et al., “Small Vessel Disease Is Associated With Altered Cerebrovascular Pulsatility but Not Resting Cerebral Blood Flow,” Journal of Cerebral Blood Flow & Metabolism 40, no. 1 (2020): 85–99, 10.1177/0271678X18803956. 30295558 PMC6928551

[49] B. Fülesdi , M. Limburg , D. Bereczki , et al., “Cerebrovascular Reactivity and Reserve Capacity in Type II Diabetes Mellitus,” Journal of Diabetes and its Complications 13, no. 4 (1999): 191–199, 10.1016/s1056-8727(99)00044-6. 10616858

[50] L. Labrecque , J. D. Smirl , and P. Brassard , “Utilization of the Repeated Squat‐Stand Model for Studying the Directional Sensitivity of the Cerebral Pressure‐Flow Relationship,” Journal of Applied Physiology 131, no. 3 (2021): 927–936, 10.1152/japplphysiol.00269.2021. 34264130

[51] D. Panidis , K. Tziomalos , G. Misichronis , et al., “Insulin Resistance and Endocrine Characteristics of the Different Phenotypes of Polycystic Ovary Syndrome: A Prospective Study,” Human Reproduction 27, no. 2 (2012): 541–549, 10.1093/humrep/der418. 22144419

[52] L. Labrecque , J. D. Smirl , Y.‐C. Tzeng , and P. Brassard , “Point/Counterpoint: We Should Take the Direction of Blood Pressure Change into Consideration for Dynamic Cerebral Autoregulation Quantification,” Journal of Cerebral Blood Flow and Metabolism: Official Journal of the International Society of Cerebral Blood Flow and Metabolism 42, no. 12 (2022): 2351–2353, 10.1177/0271678X221104868. 35619230 PMC9670010

