# Supplementary material for: Resting Cerebrovascular Haemodynamics and Dynamic Assessment of Cerebrovascular Function in Polycystic Ovary Syndrome
Source: Clin Endocrinol (Oxf). 2025 Nov 25;104(3):245–54. doi: 10.1111/cen.70061 (PMC12865755; doi:10.1111/cen.70061)
Supplement: Supplementary file 1 — Supporting Materials FINAL. [file CEN-104-245-s001.docx]

## Supplementary Materials

**MRI Acquisition**

A structural T1- magnetization prepared rapid acquisition gradient echo (MP-RAGE) scan was acquired for image registration, brain segmentation and generation of a grey matter template to mask perfusion data (1mm^3^; repetition time [TR]=2.1s; echo time [TE]=3.24ms). A multiple post label delay pseudo continuous arterial spin labelling sequence (pCASL) was used to generate perfusion maps for calculating grey matter CBF (gmCBF). Imaging parameters for the multiple post label delay pCASL sequence were: (maximum TR=5.6s; TE=11s; voxel resolution=3.4x3.4x6.0mm; tag duration=1500; post-labelling delays [PLDs]=250-3000ms in steps of 250ms; GRAPPA=2).

**MRI Processing**

Pre-processing of the pCASL sequence was completed using the Analysis of Functional NeuroImages (AFNI) software package^1^ (Available at: <http://afni.nimh.nih.gov/afni>). Following motion correction, the scan was split into separate PLDs (5 pairs of each) and the delta-M was calculated (tag minus control). For quantification, the M0 of the blood was calculated as: 0.7212 * M0 cerebrospinal fluid * exp (0.019 * [{1/T2ref} - {1/T2blood}]). Whereby M0csf is the M0 of the cerebral spinal fluid (CSF) and was taken from a CSF mask generated around a manually positioned central point in the lateral ventricles, T2ref is T2 * CSF (0.6) and T2blood is T2* of blood (0.106). The 3dNLfim command and a PCASL_Buxton signal model was used for final quantification. Gray matter cerebral blood flow values were allowed to vary between 0 and 300. The final gray matter cerebral blood flow map was thresholded by R^2^> 0.6. The individual grey matter mask generated from their structural T1 scan was used to restrict the perfusion maps to the cortical grey matter. The final grey matter cerebral blood flow (gmCBF) value was taken as the median from this restricted map.

**NVC Data Processing and Quality Control**

NVC was measured in line with recommendations from a recent consensus agreement^2^. Beat-by-beat cardiovascular and cerebrovascular data were cubic spline interpolated at 5Hz using a custom-built MATLab code (The MathWorks, Natick, MA, USA)^3–5^. All data was visually inspected for noise, aligned to the onset of the first ‘eyes open’ trial and averaged to create a single response per participant. Data were excluded from analysis if less than three of the five PCAv cycles were feasible trials, deduced by visually inspecting an initial peak from baseline, followed by a reduction in PCAv. These quality checks resulted in the exclusion of 2 women with PCOS and 3 controls from this analysis.

**Retrospective Power Analysis**

A post hoc power analysis was conducted using G*Power 3.1 to estimate the achieved power for the independent samples t-test based on the final sample sizes for each outcome variable, an alpha level of 0.05, and the calculated effect size (Cohen’s d; Supplementary Table 1).

Supplementary Table 1. Retrospective power calculations for all outcome variables

| Assessment | PCOS (n) | Control (n) | α | Effect Size | 1-β |
| --- | --- | --- | --- | --- | --- |
| gmCBF | 15 | 12 | 0.05 | 0.45 | 0.20 |
| NVC | 13 | 13 |  | 0.24 | 0.09 |
| dCA | 14 | 15 |  | 0.22 | 0.09 |
| CVR_CO2_ | 10 | 15 |  | 0.57 | 0.26 |

Abbreviations: gmCBF, grey matter cerebral blood flow; NVC, neurovascular coupling; dCA, dynamic cerebral autoregulation; CVR_CO2_, cerebrovascular reactivity to carbon dioxide.

**References**

1. Cox RW. AFNI: software for analysis and visualization of functional magnetic resonance neuroimages. *Comput Biomed Res*. 1996;29(3):162-173. doi:10.1006/cbmr.1996.0014

2. Ball JD, Hills E, Altaf A, et al. Neurovascular coupling methods in healthy individuals using transcranial doppler ultrasonography: A systematic review and consensus agreement. *J Cereb Blood Flow Metab*. 2024;44(12):1409-1429. doi:10.1177/0271678X241270452

3. Phillips AA, Chan FH, Zheng MMZ, Krassioukov AV, Ainslie PN. Neurovascular coupling in humans: Physiology, methodological advances and clinical implications. *J Cereb Blood Flow Metab*. 2016;36(4):647-664. doi:10.1177/0271678X15617954

4. Koep JL, Bond B, Barker AR, et al. Sex modifies the relationship between age and neurovascular coupling in healthy adults. *J Cereb Blood Flow Metab*. 2023;43(8):1254-1266. doi:10.1177/0271678X231167753

5. Talbot JS, Perkins DR, Dawkins TG, et al. Neurovascular coupling and cerebrovascular hemodynamics are modified by exercise training status at different stages of maturation during youth. *Am J Physiol Heart Circ Physiol*. 2023;325(3):H510-H521. doi:10.1152/ajpheart.00302.2023
